# Ethyl 3-{5-[(diethyl­amino)meth­yl]isoxazol-3-yl}-2-phenyl­pyrazolo[1,5-*a*]pyridine-5-carboxyl­ate

**DOI:** 10.1107/S1600536810004174

**Published:** 2010-03-03

**Authors:** Qing-Yang Meng, Jiong Jia, Ling Yin, Fu-Xu Zhan, Jian-Wu Wang

**Affiliations:** a School of Chemistry and Chemical Engineering, Shandong University, Jinan 250100, People’s Republic of China

## Abstract

In the title compound, C_24_H_26_N_4_O_3_, the pyrazolo[1,5-*a*]pyridine ring system makes dihedral angles of 38.130 (3) and 30.120 (3)°, respectively, with the isoxazole and phenyl rings. In the crystal, two mol­ecules are linked by a pair of C—H⋯N hydrogen bonds, forming a centrosymmetric dimer. A weak intra­molecular C—H⋯O inter­action is also present.

## Related literature

For the bioactivity of pyrazolo[1,5-a]pyridine and isoxazole derivatives, see: Cuny *et al.* (2008 ▶); Ge *et al.* (2009 ▶); Johns *et al.* (2005 ▶); Lanig *et al.* (2001 ▶); Lee *et al.* (2009 ▶). For the synthesis of ethyl 3-(5-((methyl­sulfon­yloxy)meth­yl)isoxazol-3-yl)-2-phenyl-*H*-pyrazolo[1,5-a]pyridine-5-carboxyl­ate, see: Meng *et al.* (2010 ▶). 
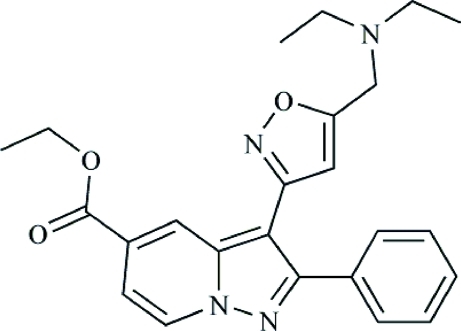


## Experimental

### 

#### Crystal data

C_24_H_26_N_4_O_3_
*M* *_r_* = 418.49
Triclinic, 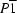

*a* = 6.1250 (7) Å
*b* = 13.1425 (16) Å
*c* = 13.7139 (16) Å
α = 93.600 (2)°
β = 95.514 (2)°
γ = 95.637 (2)°
*V* = 1090.6 (2) Å^3^
*Z* = 2
Mo *K*α radiation
μ = 0.09 mm^−1^
*T* = 298 K
0.10 × 0.10 × 0.10 mm

#### Data collection

Bruker APEXII CCD area-detector diffractometer
Absorption correction: multi-scan (*SADABS*; Sheldrick, 2003 ▶) *T*_min_ = 0.992, *T*_max_ = 0.992
5453 measured reflections
3800 independent reflections
2842 reflections with *I* > 2σ(*I*)
*R*_int_ = 0.014

#### Refinement

*R*[*F* ^2^ > 2σ(*F* ^2^)] = 0.061
*wR*(*F* ^2^) = 0.187
*S* = 1.07
3800 reflections
281 parameters
1 restraint
H-atom parameters constrained
Δρ_max_ = 0.75 e Å^−3^
Δρ_min_ = −0.35 e Å^−3^

### 

Data collection: *APEX2* (Bruker, 2004 ▶); cell refinement: *SAINT-Plus* (Bruker, 2001 ▶); data reduction: *SAINT-Plus*; program(s) used to solve structure: *SHELXS97* (Sheldrick, 2008 ▶); program(s) used to refine structure: *SHELXL97* (Sheldrick, 2008 ▶); molecular graphics: *SHELXTL* (Sheldrick, 2008 ▶); software used to prepare material for publication: *SHELXTL*.

## Supplementary Material

Crystal structure: contains datablocks global, I. DOI: 10.1107/S1600536810004174/is2515sup1.cif

Structure factors: contains datablocks I. DOI: 10.1107/S1600536810004174/is2515Isup2.hkl

Additional supplementary materials:  crystallographic information; 3D view; checkCIF report

## Figures and Tables

**Table 1:** Hydrogen-bond geometry (Å, °)

*D*—H⋯*A*	*D*—H	H⋯*A*	*D*⋯*A*	*D*—H⋯*A*
C7—H7⋯N2^i^	0.93	2.54	3.456 (3)	169
C22—H22*A*⋯O3	0.97	2.52	3.218 (3)	129

Symmetry code: (i) 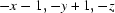.

**Fig. 1.**
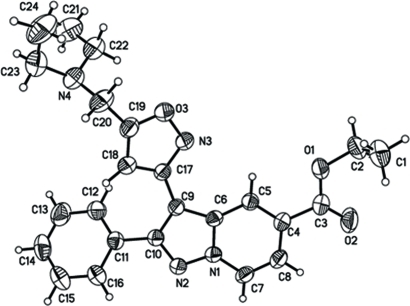
A view of the title compound, with the atom-labelling scheme. Displacement ellipsoids are drawn at the 50% probability level.

## supplementary crystallographic information

### Comment

Pyrazolo[1,5-a]pyridine derivatives have been intensively investigated due to their widespread biological activities (Cuny *et al.*, 2008; Johns *et al.*, 2005; Lanig *et al.*, 2001). Thus, it is necessary to further widen the system of application of heterocycle compounds. Recently, an interesting intramolecular condensation of α,β-unsaturated esters with aldehydes has been discovered, leading to a series of pyrazolo[1,5-a]pyridines under mild conditions (Ge *et al.*, 2009). Moreover, it is well known that many compounds with isoxazole core show potent antitumor activities (Lee *et al.*, 2009). It is therefore worth trying to incorporate isoxazole core into pyrazolo[1,5-a]pyridine scaffold to improve the biological activity (Meng *et al.*, 2010). Herein, a novel heterocycle compound has been obtained and its molecular structure is depicted (Fig. 1).

### Experimental

To a solution of ethyl 3-{5-[(methylsulfonyloxy)methyl]isoxazol-3-yl}-2-phenyl *H*-pyrazolo[1,5-a]pyridine-5-carboxylate (Meng *et al.*, 2010) (0.33 g, 0.75 mmol) in THF (20 ml) was added diethylamine (0.22 ml, 2.25 mmol). The mixture was stirred for 12 h. Water and dichloromethane were added in turn and stirred, and layers were separated. The aqueous layer was back-extracted with dichloromethane. The combined organics were washed with brine, dried over sodium sulfate, filtered and concentrated. The residue was purified by column chromatography (yield 89%). The crystals of (I) were obtained from a hexane-ethyl acetate-dichloromethane (3:1:1, *v*/*v*/*v*) solution by slow evaporation at room temperature (m.p. 363–364 K).

### Refinement

H atoms were refined using a riding model, with C—H = 0.93–0.97 Å, and with *U*_iso_(H) = 1.2*U*_eq_(C) or 1.5U_eq_(methyl C). In addition, a rigid-body restraint '*DELU*' was applied for atoms C21 and C22.

### Crystal data

**Table:**

C_24_H_26_N_4_O_3_	*Z* = 2
*M**_r_* = 418.49	*F*(000) = 444
Triclinic, *P*1	*D*_x_ = 1.274 Mg m^−^^3^
Hall symbol: -P 1	Mo *K*α radiation, λ = 0.71073 Å
*a* = 6.1250 (7) Å	Cell parameters from 1181 reflections
*b* = 13.1425 (16) Å	θ = 2.5–25.5°
*c* = 13.7139 (16) Å	µ = 0.09 mm^−^^1^
α = 93.600 (2)°	*T* = 298 K
β = 95.514 (2)°	Block, colorless
γ = 95.637 (2)°	0.10 × 0.10 × 0.10 mm
*V* = 1090.6 (2) Å^3^	

### Data collection

**Table:**

Bruker APEXII CCD area-detector diffractometer	3800 independent reflections
Radiation source: fine-focus sealed tube	2842 reflections with *I* > 2σ(*I*)
graphite	*R*_int_ = 0.014
Detector resolution: 0 pixels mm^-1^	θ_max_ = 25.0°, θ_min_ = 1.5°
φ and ω scans	*h* = −7→7
Absorption correction: multi-scan (*SADABS*; Sheldrick, 2003)	*k* = −15→13
*T*_min_ = 0.992, *T*_max_ = 0.992	*l* = −14→16
5453 measured reflections	

### Refinement

**Table:**

Refinement on *F*^2^	Primary atom site location: structure-invariant direct methods
Least-squares matrix: full	Secondary atom site location: difference Fourier map
*R*[*F*^2^ > 2σ(*F*^2^)] = 0.061	Hydrogen site location: inferred from neighbouring sites
*wR*(*F*^2^) = 0.187	H-atom parameters constrained
*S* = 1.07	*w* = 1/[σ^2^(*F*_o_^2^) + (0.0993*P*)^2^ + 0.4336*P*] where *P* = (*F*_o_^2^ + 2*F*_c_^2^)/3
3800 reflections	(Δ/σ)_max_ = 0.004
281 parameters	Δρ_max_ = 0.75 e Å^−^^3^
1 restraint	Δρ_min_ = −0.34 e Å^−^^3^

### Special details

**Table:**

Geometry. All esds (except the esd in the dihedral angle between two l.s. planes) are estimated using the full covariance matrix. The cell esds are taken into account individually in the estimation of esds in distances, angles and torsion angles; correlations between esds in cell parameters are only used when they are defined by crystal symmetry. An approximate (isotropic) treatment of cell esds is used for estimating esds involving l.s. planes.
Refinement. Refinement of *F*^2^ against ALL reflections. The weighted *R*-factor wR and goodness of fit *S* are based on *F*^2^, conventional *R*-factors *R* are based on *F*, with *F* set to zero for negative *F*^2^. The threshold expression of *F*^2^ > σ(*F*^2^) is used only for calculating *R*-factors(gt) etc. and is not relevant to the choice of reflections for refinement. *R*-factors based on *F*^2^ are statistically about twice as large as those based on *F*, and *R*- factors based on ALL data will be even larger.

### Fractional atomic coordinates and isotropic or equivalent isotropic displacement parameters (Å^2^)

**Table:**

	*x*	*y*	*z*	*U*_iso_*/*U*_eq_	
C1	0.2802 (5)	−0.0313 (2)	0.0758 (3)	0.0669 (9)	
H1A	0.4008	−0.0724	0.0722	0.100*	
H1B	0.2200	−0.0200	0.0106	0.100*	
H1C	0.1680	−0.0663	0.1094	0.100*	
C2	0.3600 (5)	0.0686 (2)	0.1300 (2)	0.0585 (8)	
H2A	0.4219	0.0570	0.1957	0.070*	
H2B	0.4758	0.1031	0.0968	0.070*	
C3	0.1430 (4)	0.19374 (19)	0.06317 (19)	0.0441 (6)	
C4	−0.0421 (4)	0.25659 (18)	0.07983 (17)	0.0393 (6)	
C5	−0.1456 (4)	0.25212 (18)	0.16402 (17)	0.0366 (5)	
H5	−0.1060	0.2073	0.2109	0.044*	
C6	−0.3125 (4)	0.31605 (17)	0.17869 (16)	0.0346 (5)	
C7	−0.2687 (4)	0.3840 (2)	0.02118 (18)	0.0451 (6)	
H7	−0.3130	0.4270	−0.0266	0.054*	
C8	−0.1074 (5)	0.3235 (2)	0.00756 (18)	0.0470 (6)	
H8	−0.0380	0.3255	−0.0498	0.056*	
C9	−0.4470 (4)	0.33567 (17)	0.25335 (16)	0.0358 (5)	
C10	−0.5678 (4)	0.41490 (18)	0.22118 (17)	0.0366 (5)	
C11	−0.7208 (4)	0.47407 (18)	0.27307 (18)	0.0387 (6)	
C12	−0.6795 (5)	0.5013 (2)	0.3729 (2)	0.0556 (7)	
H12	−0.5587	0.4790	0.4087	0.067*	
C13	−0.8164 (6)	0.5616 (3)	0.4199 (2)	0.0686 (9)	
H13	−0.7867	0.5799	0.4868	0.082*	
C14	−0.9967 (6)	0.5946 (2)	0.3677 (3)	0.0675 (9)	
H14	−1.0897	0.6345	0.3993	0.081*	
C15	−1.0381 (5)	0.5682 (2)	0.2687 (3)	0.0610 (8)	
H15	−1.1597	0.5904	0.2335	0.073*	
C16	−0.9014 (4)	0.50908 (19)	0.2210 (2)	0.0464 (6)	
H16	−0.9301	0.4925	0.1538	0.056*	
C17	−0.4706 (4)	0.27444 (18)	0.33801 (17)	0.0369 (5)	
C18	−0.6611 (4)	0.2496 (2)	0.38597 (18)	0.0437 (6)	
H18	−0.7981	0.2739	0.3743	0.052*	
C19	−0.6021 (5)	0.1835 (2)	0.45177 (19)	0.0467 (6)	
C20	−0.7261 (5)	0.1236 (2)	0.5216 (2)	0.0582 (8)	
H20A	−0.8790	0.1091	0.4945	0.070*	
H20B	−0.6655	0.0586	0.5283	0.070*	
C21	−0.4753 (6)	0.2470 (3)	0.7671 (2)	0.0775 (10)	
H21A	−0.3289	0.2451	0.7985	0.116*	
H21B	−0.5810	0.2223	0.8097	0.116*	
H21C	−0.4972	0.3162	0.7534	0.116*	
C22	−0.5053 (6)	0.1792 (3)	0.6711 (2)	0.0719 (9)	
H22A	−0.3953	0.2038	0.6293	0.086*	
H22B	−0.4793	0.1099	0.6855	0.086*	
C23	−0.9023 (6)	0.1347 (3)	0.6722 (3)	0.0747 (10)	
H23A	−0.9082	0.1799	0.7304	0.090*	
H23B	−1.0389	0.1367	0.6305	0.090*	
C24	−0.8942 (7)	0.0293 (3)	0.7027 (3)	0.0898 (12)	
H24A	−1.0212	0.0102	0.7361	0.135*	
H24B	−0.7628	0.0263	0.7462	0.135*	
H24C	−0.8935	−0.0170	0.6458	0.135*	
N1	−0.3662 (3)	0.38084 (15)	0.10689 (14)	0.0374 (5)	
N2	−0.5209 (3)	0.44225 (16)	0.13189 (15)	0.0419 (5)	
N3	−0.3039 (4)	0.22794 (19)	0.37371 (17)	0.0572 (7)	
N4	−0.7175 (4)	0.1771 (2)	0.61889 (18)	0.0590 (7)	
O1	0.1826 (3)	0.13349 (14)	0.13639 (14)	0.0520 (5)	
O2	0.2449 (4)	0.19763 (17)	−0.00730 (16)	0.0689 (6)	
O3	−0.3882 (4)	0.16863 (16)	0.44714 (14)	0.0633 (6)	

### Atomic displacement parameters (Å^2^)

**Table:**

	*U*^11^	*U*^22^	*U*^33^	*U*^12^	*U*^13^	*U*^23^
C1	0.065 (2)	0.0539 (18)	0.085 (2)	0.0132 (15)	0.0186 (17)	−0.0004 (16)
C2	0.0485 (16)	0.0600 (18)	0.0696 (19)	0.0210 (14)	0.0073 (14)	−0.0002 (15)
C3	0.0457 (14)	0.0414 (13)	0.0458 (15)	0.0024 (11)	0.0136 (12)	−0.0025 (11)
C4	0.0429 (14)	0.0386 (13)	0.0365 (13)	0.0026 (11)	0.0078 (11)	−0.0012 (10)
C5	0.0392 (13)	0.0350 (12)	0.0363 (12)	0.0049 (10)	0.0056 (10)	0.0038 (10)
C6	0.0401 (13)	0.0329 (12)	0.0311 (12)	0.0036 (10)	0.0039 (10)	0.0046 (9)
C7	0.0555 (16)	0.0502 (15)	0.0315 (13)	0.0082 (12)	0.0076 (11)	0.0076 (11)
C8	0.0549 (16)	0.0543 (15)	0.0342 (13)	0.0087 (13)	0.0132 (12)	0.0043 (11)
C9	0.0400 (13)	0.0356 (12)	0.0332 (12)	0.0069 (10)	0.0063 (10)	0.0037 (10)
C10	0.0384 (13)	0.0367 (12)	0.0351 (12)	0.0061 (10)	0.0039 (10)	0.0031 (10)
C11	0.0407 (13)	0.0335 (12)	0.0430 (14)	0.0048 (10)	0.0078 (11)	0.0030 (10)
C12	0.0641 (18)	0.0588 (17)	0.0466 (16)	0.0237 (14)	0.0045 (13)	−0.0007 (13)
C13	0.086 (2)	0.066 (2)	0.0558 (18)	0.0193 (18)	0.0184 (17)	−0.0086 (15)
C14	0.065 (2)	0.0577 (18)	0.087 (2)	0.0223 (16)	0.0309 (18)	−0.0025 (17)
C15	0.0444 (16)	0.0529 (17)	0.087 (2)	0.0141 (13)	0.0048 (15)	0.0008 (16)
C16	0.0408 (14)	0.0412 (14)	0.0570 (16)	0.0055 (11)	0.0037 (12)	0.0020 (12)
C17	0.0440 (13)	0.0370 (12)	0.0314 (12)	0.0096 (10)	0.0066 (10)	0.0034 (10)
C18	0.0437 (14)	0.0512 (15)	0.0383 (13)	0.0078 (12)	0.0073 (11)	0.0102 (11)
C19	0.0552 (16)	0.0468 (14)	0.0409 (14)	0.0075 (12)	0.0145 (12)	0.0065 (11)
C20	0.078 (2)	0.0523 (16)	0.0456 (16)	−0.0024 (15)	0.0160 (14)	0.0106 (13)
C21	0.092 (3)	0.083 (2)	0.0550 (19)	−0.003 (2)	0.0054 (18)	0.0036 (17)
C22	0.064 (2)	0.091 (2)	0.068 (2)	0.0180 (18)	0.0230 (17)	0.0296 (18)
C23	0.065 (2)	0.091 (3)	0.075 (2)	0.0170 (18)	0.0260 (18)	0.0182 (19)
C24	0.116 (3)	0.078 (2)	0.078 (2)	−0.013 (2)	0.034 (2)	0.022 (2)
N1	0.0417 (11)	0.0386 (11)	0.0334 (10)	0.0087 (9)	0.0044 (8)	0.0060 (8)
N2	0.0459 (12)	0.0455 (12)	0.0373 (11)	0.0144 (10)	0.0060 (9)	0.0080 (9)
N3	0.0639 (15)	0.0715 (16)	0.0474 (13)	0.0290 (13)	0.0242 (11)	0.0290 (12)
N4	0.0586 (15)	0.0684 (16)	0.0547 (15)	0.0082 (12)	0.0184 (12)	0.0185 (12)
O1	0.0531 (11)	0.0529 (11)	0.0554 (11)	0.0202 (9)	0.0161 (9)	0.0066 (9)
O2	0.0720 (14)	0.0770 (15)	0.0680 (14)	0.0234 (12)	0.0390 (12)	0.0133 (11)
O3	0.0735 (14)	0.0733 (14)	0.0565 (12)	0.0356 (11)	0.0274 (10)	0.0345 (10)

### Geometric parameters (Å, °)

**Table:**

C1—C2	1.482 (4)	C14—C15	1.373 (5)
C1—H1A	0.9600	C14—H14	0.9300
C1—H1B	0.9600	C15—C16	1.379 (4)
C1—H1C	0.9600	C15—H15	0.9300
C2—O1	1.451 (3)	C16—H16	0.9300
C2—H2A	0.9700	C17—N3	1.311 (3)
C2—H2B	0.9700	C17—C18	1.415 (3)
C3—O2	1.201 (3)	C18—C19	1.340 (4)
C3—O1	1.336 (3)	C18—H18	0.9300
C3—C4	1.493 (4)	C19—O3	1.350 (3)
C4—C5	1.371 (3)	C19—C20	1.494 (4)
C4—C8	1.420 (4)	C20—N4	1.462 (4)
C5—C6	1.407 (3)	C20—H20A	0.9700
C5—H5	0.9300	C20—H20B	0.9700
C6—N1	1.379 (3)	C21—C22	1.528 (5)
C6—C9	1.402 (3)	C21—H21A	0.9600
C7—C8	1.347 (4)	C21—H21B	0.9600
C7—N1	1.370 (3)	C21—H21C	0.9600
C7—H7	0.9300	C22—N4	1.419 (4)
C8—H8	0.9300	C22—H22A	0.9700
C9—C10	1.404 (3)	C22—H22B	0.9700
C9—C17	1.464 (3)	C23—C24	1.476 (5)
C10—N2	1.347 (3)	C23—N4	1.487 (4)
C10—C11	1.480 (3)	C23—H23A	0.9700
C11—C12	1.386 (4)	C23—H23B	0.9700
C11—C16	1.391 (3)	C24—H24A	0.9600
C12—C13	1.384 (4)	C24—H24B	0.9600
C12—H12	0.9300	C24—H24C	0.9600
C13—C14	1.379 (5)	N1—N2	1.358 (3)
C13—H13	0.9300	N3—O3	1.417 (3)
			
C2—C1—H1A	109.5	C15—C16—H16	119.9
C2—C1—H1B	109.5	C11—C16—H16	119.9
H1A—C1—H1B	109.5	N3—C17—C18	111.6 (2)
C2—C1—H1C	109.5	N3—C17—C9	119.6 (2)
H1A—C1—H1C	109.5	C18—C17—C9	128.7 (2)
H1B—C1—H1C	109.5	C19—C18—C17	105.3 (2)
O1—C2—C1	111.2 (2)	C19—C18—H18	127.4
O1—C2—H2A	109.4	C17—C18—H18	127.4
C1—C2—H2A	109.4	C18—C19—O3	109.4 (2)
O1—C2—H2B	109.4	C18—C19—C20	133.0 (3)
C1—C2—H2B	109.4	O3—C19—C20	117.5 (2)
H2A—C2—H2B	108.0	N4—C20—C19	113.1 (2)
O2—C3—O1	124.2 (2)	N4—C20—H20A	109.0
O2—C3—C4	123.9 (3)	C19—C20—H20A	109.0
O1—C3—C4	111.9 (2)	N4—C20—H20B	109.0
C5—C4—C8	120.0 (2)	C19—C20—H20B	109.0
C5—C4—C3	121.2 (2)	H20A—C20—H20B	107.8
C8—C4—C3	118.8 (2)	C22—C21—H21A	109.5
C4—C5—C6	119.3 (2)	C22—C21—H21B	109.5
C4—C5—H5	120.4	H21A—C21—H21B	109.5
C6—C5—H5	120.4	C22—C21—H21C	109.5
N1—C6—C9	105.8 (2)	H21A—C21—H21C	109.5
N1—C6—C5	118.2 (2)	H21B—C21—H21C	109.5
C9—C6—C5	136.0 (2)	N4—C22—C21	113.7 (3)
C8—C7—N1	118.6 (2)	N4—C22—H22A	108.8
C8—C7—H7	120.7	C21—C22—H22A	108.8
N1—C7—H7	120.7	N4—C22—H22B	108.8
C7—C8—C4	120.7 (2)	C21—C22—H22B	108.8
C7—C8—H8	119.6	H22A—C22—H22B	107.7
C4—C8—H8	119.6	C24—C23—N4	116.8 (3)
C6—C9—C10	104.9 (2)	C24—C23—H23A	108.1
C6—C9—C17	125.0 (2)	N4—C23—H23A	108.1
C10—C9—C17	129.6 (2)	C24—C23—H23B	108.1
N2—C10—C9	112.2 (2)	N4—C23—H23B	108.1
N2—C10—C11	118.0 (2)	H23A—C23—H23B	107.3
C9—C10—C11	129.7 (2)	C23—C24—H24A	109.5
C12—C11—C16	118.7 (2)	C23—C24—H24B	109.5
C12—C11—C10	120.9 (2)	H24A—C24—H24B	109.5
C16—C11—C10	120.3 (2)	C23—C24—H24C	109.5
C13—C12—C11	120.6 (3)	H24A—C24—H24C	109.5
C13—C12—H12	119.7	H24B—C24—H24C	109.5
C11—C12—H12	119.7	N2—N1—C7	124.3 (2)
C14—C13—C12	120.1 (3)	N2—N1—C6	112.60 (19)
C14—C13—H13	119.9	C7—N1—C6	123.1 (2)
C12—C13—H13	119.9	C10—N2—N1	104.49 (18)
C15—C14—C13	119.6 (3)	C17—N3—O3	104.9 (2)
C15—C14—H14	120.2	C22—N4—C20	111.2 (3)
C13—C14—H14	120.2	C22—N4—C23	114.6 (3)
C14—C15—C16	120.7 (3)	C20—N4—C23	110.5 (3)
C14—C15—H15	119.6	C3—O1—C2	117.7 (2)
C16—C15—H15	119.6	C19—O3—N3	108.85 (19)
C15—C16—C11	120.3 (3)		

### Hydrogen-bond geometry (Å, °)

**Table:**

*D*—H···*A*	*D*—H	H···*A*	*D*···*A*	*D*—H···*A*
C7—H7···N2^i^	0.93	2.54	3.456 (3)	169
C22—H22A···O3	0.97	2.52	3.218 (3)	129

Symmetry codes: (i) −*x*−1, −*y*+1, −*z*.
